# The Bioactive Potential of Functional Products and Bioavailability of Phenolic Compounds

**DOI:** 10.3390/foods9070953

**Published:** 2020-07-18

**Authors:** Cristina Monica Dabulici, Ionela Sârbu, Emanuel Vamanu

**Affiliations:** 1 Faculty of Biotechnology, University of Agronomic Science and Veterinary Medicine, 59 Marasti blvd, 1 district, 011464 Bucharest, Romania; dcm.cristina.cm@gmail.com; 2 Department of Genetics, ICUB-Research Institute of the University of Bucharest, 36-46 Bd. M. Kogalniceanu, 5th District, 050107 Bucharest, Romania; ionela24avram@yahoo.com

**Keywords:** yeast, antioxidant, cytotoxicity, bioavailability, viability

## Abstract

The expression of bioactivity depends on the assimilation of different classes of natural substances (e.g., phenolic compounds) in vivo. Six functional extracts (*Aspalathus linearis*, leaves; *Paullinia cupana*, seeds; *Aristotelia chilensis*, berries; *Ilex paraguariensis*, leaves; *Syzygium aromaticum*, cloves, and wild berries) were analyzed in vitro and in vivo as an alternative to alleviating pathologies associated with oxidative stress (proliferation of cancer cells). The purpose of this research was to evaluate the in vitro and in vivo antioxidant and cytotoxic potential of hydroalcoholic solutions, in addition to the assimilation capacity of bioactive components in *Saccharomyces boulardii* cells. In vivo antioxidant capacity (critical point value) was correlated with the assimilation ratio of functional compounds. The results of in vitro antioxidant activities were correlated with the presence of quercetin (4.67 ± 0.27 mg/100 mL) and chlorogenic acid (14.38 ± 0.29 mg/100 mL) in *I. paraguariensis*. Bioassimilation of the main nutraceutical components depended on the individual sample. Phenolic acid levels revealed the poor assimilation of the main components, which could be associated with cell viability to oxidative stress.

## 1. Introduction

Most phenolic compounds are assimilated at the time of transit to the small intestine [1], but some are biotransformed in the colon under the action of the microbiota. As a result, the biological value may be altered because of new compounds resulting after the fermentative action of the microbial pattern. Some portion of these compounds may be completely degraded, as observed for compounds with a high molecular weight (e.g., curcumin) [2]. Despite being known for their antimicrobial effect, certain compounds (such as phenolics) can be assimilated (bioaccumulation) by certain yeast strains, such as *Rhodotorula mucilaginosa* [3]. This bioavailability process has been partially observed for gallic acid. Its use as a carbon source explains the biological response determined by the consumption of these functional molecules [4]. The determination of bioavailability [5] is much more significant in vivo as an indicator of cell absorption because for phenolic compounds the accumulation is difficult to evaluate [6].

The use of a carrier vehicle for the delivery of phenolic compounds increases the in vivo bioavailability [7] and reduces the yield of biotransformations that limit the bioactivity expression. For example, gallic acid has no stability in the fermentative action of the microbiota, but resistance to the action of oxidative stress is mediated through this compound [8]. Its action is limited by the amount that is absorbed into the small intestine, because the remaining amount is inaccessible in vivo [9].

The use of probiotic yeast biomass (such as *Saccharomyces boulardii*) for assimilating these compounds increases bioavailability, and the biological action may be performed in such cases via direct action on the microbial pattern in the colon [2,10]. This process is equivalent to biotransformation into an intermediate compound. Modulation of the microbiological response can be achieved both by restoring eubiosis and promoting the synthesis of compounds with a biomarker role (e.g., short-chain fatty acids) [4]. The metabolism of such compounds has demonstrated the presence of strains belonging to the genus *Bacteroides* and/or the phylum Firmicutes, while the synthesis of compounds has had positive effects on human well-being [11].

New sources of biologically-active compounds have been identified after the determination of bioactivity and the chemical characterization of the natural substrate represented by medicinal herbs [12]. The quantity of functional compounds (such as polyphenolcarboxylic acids) depends on the vegetal material [13], the product conditioning mode, the climatic conditions, the soil composition, and the vegetative stage of the plant [14]. The presence of different bioactive compounds in a large quantity has been correlated with increased resistance to the action of free radicals, which increases life expectancy [15].

A comparison of the biological capacities (such as antioxidant and cytotoxic activities) of different products provides a valid tool to determine their health benefits [16]; functional products have greater benefits because of their antioxidant effect [17]. However, the total antioxidant capacity is the result of the presence of several classes of compounds (such as vitamin C, flavonoids, phenolic acids, and anthocyanins), which affect human health differently. Therefore, in vitro determination does not reflect all of the biological activity [18]. The contribution of individual compounds and/or effects in vivo should be examined through models that provide an image of the interaction and bioassimilation of these molecules [19]. Bioassimilation could be defined as the results of uptake of the bioaccessible phenolic fraction by the yeast cells by mechanisms of transmembrane absorption [20].

Therefore, this study aimed to compare the antioxidant and cytotoxic potentials of hydroalcoholic extracts of the following: (1) *Aspalathus linearis* (rooibos; high in antioxidants, but low tannin content and without caffeine; used for protection against stroke, heart disease, and cancer) [21,22]; (2) *Paullinia cupana* (guarana: used for physical, mental, and cancer-related fatigue with no significant side effects) [23]; (3) *Aristotelia chilensis (Mol.)* Stuntz (maqui; different therapeutic properties based on levels of antioxidants) [24]; (4) *Ilex paraguariensis* A. St.-Hil. (yerba mate; source of biological compounds for the nutraceutical industry) [25]; (5) *Syzygium aromaticum* (L.) Merr. & L.M.Perry (cloves; food preservative and different medicinal purposes) [26]; and (6) wild berry (*Rosa canina, Rubus idaeus*, and *Vaccinium myrtillus*—1:1:1; which are relevant to the prevention of degenerative diseases) [27]. The effect on probiotic yeast, *S. boulardii,* was examined by determining the assimilation capacity of cells (enriched biomass) as their in vivo bioassimilation and antioxidant capacity.

## 2. Materials and Methods

### 2.1. Samples

Six dried samples (*A. linearis*, leaves; *P. cupana*, seeds; *A. chilensis*, berries; *I. paraguariensis*, leaves; *S. aromaticum*, cloves, and wild berries) were tested and selected because of their use as alternatives for alleviating pathologies associated with oxidative stress. All samples were purchased from local shops (e.g., Kaufland, Romania) and were dried in an air oven (Memmert oven UFB400) at 40 °C until a constant weight was obtained. The samples (10 g) were mixed with 50% ethanol (to determine a balance between the bioactive compounds present in the extracts) and stored for 48 h in the dark until the mixture was filtered under vacuum. A quantity of 100 g for each sample and 1000 mL solvent was used. The solutions were stored in brown bottles [8].

### 2.2. Determination of Bioactive Compounds

The experiments were performed with high-pressure liquid chromatograph (HPLC), ELITE-LaChrom (Merck-Hitachi, Tokyo, Japan), with DAD (Diode-Array Detection) detector analytic scales (KERN 770). The chromatographic column of stainless steel comprised the stationary stage of octadecylsilane (Inertsil ODS-3 250 * 4.6  mm * 5  μm); mobile stages—mobile stage A, phosphoric acid/water, pH = 2.5 and mobile stage B, methanol. Flow rate of the mobile stage was 1.0  mL/min; elution type: with linear composition gradient of the mobile stage. UV detection was ʎ = 330 nm; temperature of column oven, 40 °C; injection volume, 20  μL. After the chromatographic system was balanced, the basic line was a straight line and the reference solution was injected. The differences between successive determinations were limited to a maximum of 2%. After the injection of the test solutions, we registered the chromatograms.

Reference solutions (mixtures of 10  μg/mL), were chlorogenic acid, caffeic acid, coumaric acid, ferulic acid, rosemarinic acid, vanillic acid, luteolin 7-glycoside, rutin, apigenin 7-glycoside, luteolin, apigenin, quercetin, quercetin 3-rhamnoside, kaempferol, kaempferol 3-rhamnoside, kaempferol 3-rutinoside, kaempferol 7-glucoside, kaempferol 3-galactoside, catechin, myricetin, myricetin 3-glucoside, and pyrogallol [28,29]. Other reagents used in the determinations were methanol, orthophosphoric acid, ultrapure water, absolute ethanol, and solvent ethanol/water (50:50, *v/v*). For the sample solution, in a measuring bottle of 50  mL, we introduced 0.5000  g of sample powder, adding around 40 mL of solvent, and performing ultrasonication for 30 min at 40 °C. Supplements to 50 mL with the same solvent and filters were added.

The content in the compounds of interest was calculated using the formula compound % = [(A_p_ × C_e_) × A_e_] × (50/G) × 100, where A_p_ is the range of the compound’s peak “i” in sample solution, A_e_ is the range of the compound’s peak “i” in the reference solution, C_e_ is the concentration of compound “i” in the reference solution (mg/mL), G is the quantity of processed sample (mg), and 50 is the correction coefficient [28,29].

### 2.3. Assessment of the In Vitro Antioxidant Potential

The antioxidant potential of all hydroalcoholic extracts was assessed by determining the DPPH (2,2-diphenyl-1-picrylhydrazyl) scavenging activity [30] and the chelating activity [31].

In the case of DPPH scavenging activity, the reaction mixture was 0.1 mL sample, 0.49 mL ethanol, and 0.39 mL DPPH (1 mM). The mixture was kept for 30 min in a dark place. The absorbance of the final mixture was measured at 517 nm against the blank.

In the case of chelating activity, the reaction mixture was 1 mL of sample mixed with 50 μL of 2 mM FeCl_2_. After 5 min, 0.2 mL of 5 mM ferrozine solution was added. The mixture was kept at room temperature for 10 min. The absorbance of the final mixture was measured at 562 nm against the blank.

Ascorbic acid (1 mg/mL) and EDTA (Ethylenediaminetetraacetic acid, 0.5 mg/mL) were used as controls, and the remaining activities were calculated using the following formula: $\frac{Abs_{M}-Abs_{P}}{Abs_{M}}$ × 100 [32], where Abs_M_ is the absorbance of the control and Abs_P_ is the absorbance of the sample.

### 2.4. Determination of the In Vivo Antioxidant Potential

The antioxidant potential in vivo was evaluated by a modified protocol [2]. A strain of *Saccharomyces boulardii*, a probiotic yeast obtained from the University of Lille, Lille, France, was used. The biomass was obtained using YPG medium (Yeast Extract–Peptone–Glucose; 2% glucose, 2% peptone, and 1% yeast extract) and further cultivated in a lab shaker incubator at 30 °C, for 48 h, at 150 rpm. The yeast cells were separated through centrifugation (4500× *g*, 5 min). The reaction mixture was 0.1 mL sample, 0.1 mL yeast cells in a sterile saline solution, and 0.2 mL H_2_O_2._ The mixture was made in 100-well honeycomb microplates (sterilized by gamma radiation) and incubated at 30 °C for 1 h using Bioscreen C MBR (Oy Growth Curves Ab Ltd., Helsinki, Finland). The critical concentration was defined as the cross between the viability and mortality lines (different concentrations of H_2_O_2_ (%)—0.05, 0.1, 0.3, 0.5, 1, 2, and 3), and expressed as a critical point (%). The untreated sample was used as a control for critical point determination. The tests were made until and after bioactive compound assimilation. Finally, the antioxidant potential in vivo was quantified as a percentage value that resulted after the bioactive compound assimilation. The results were calculated in comparison with a control that was realized at the same critical point for each sample but without an extract in the reaction mixture.

### 2.5. Bioavailability Index Quantification

The bioavailability index was determined based on the method presented by Celep et al. (2018) [33], with some modifications, using the same *S. boulardii* strain. The extract (1 mg/mL), was added to the culture media via filtration through sterile Milllipore membrane (0.22 µm) and inserted in the temperature-controlled orbital shaker (LabTech) in an 80 mL Duran bottle (sterile-venting membrane screw caps; 0.2 µm (ePTFE membrane). Chlorogenic acid (1 mg/mL) was used as a control. The bioavailability index (BI) was calculated using the following formula: $\frac{\mathrm{quantity}\;\mathrm{of}\;\mathrm{absorbed}\;\mathrm{phenolics}}{\mathrm{quantity}\;\mathrm{of}\;\mathrm{total}\;\mathrm{phenolics}}$ × 100 [2,33], where the quantity of absorbed phenolics was estimated after the biomass was freeze-dried and the presence of total phenolics was determined as presented in Section 2.2.

### 2.6. Evaluation of Cytotoxicity

The cytotoxic effect of hydroalcoholic extracts was assessed by measuring HCT-8 and *S. boulardii* cell viability using the Vybrant^®^ MTT (3-(4,5-dimethylthiazol-2-yl)-2,5-diphenyltetrazolium bromide) Cell Proliferation Assay Kit. The human epithelial HCT-8 cell line isolated from ileocecal colorectal adenocarcinoma, passage 18, was cultivated in RPMI 1640 (Sigma, St. Louis, MO, USA) supplied with 10% fetal bovine serum (FBS; Biochrom, Berlin, Germany) in polystyrene 96-well plates at 37 °C, 5% CO_2_ until they reached a 60% confluence; *S. boulardii* strain was cultured in YPG at 30 °C for 24 h, 120 rpm.

The medium was removed and the cells were incubated for 24 h in fresh media with extracts in concentrations of 10% and 1%. After the incubation, the medium was removed and the cells were washed one time with warm PBS (phosphate-buffered saline) and incubated with MTT solution for 2.5 h (human cell line) and 4 h (yeast strain).

The dye was solubilized with DMSO (Dimethyl sulfoxide) and the plate was read at 540 nm using Synergy HTX (Biotek, Winooski, VT, USA). All of the samples were performed in triplicate. Cell viability was calculated according to the formula: % cell survival = (mean sample absorbance/mean control absorbance) × 100. An untreated sample with extracts (only with 5% and 0.5% ethanol, respectively) was used as control [34].

### 2.7. Statistical Analysis

All of the parameters investigated were evaluated in a minimum of three independent determinations, and the results were expressed as the mean ± standard deviation (SD). The mean and SD values were calculated using the IBM SPSS Statistics 23 software package (IBM Corporation, Armonk, NY, USA). The significance level for the calculations was set as follows: significant, *p* ≤ 0.05; very significant, *p* ≤ 0.01; and highly significant, *p* ≤ 0.001, using the normal distribution of the variables. The differences were analyzed by ANOVA followed by a Tukey post hoc analysis. Analysis and correlation of the experimental data were conducted with the IBM SPSS Statistics software package (IBM Corporation, Armonk, NY, USA) [35].

## 3. Results

### 3.1. Determination of Bioactive Compounds

**Figure 1:**
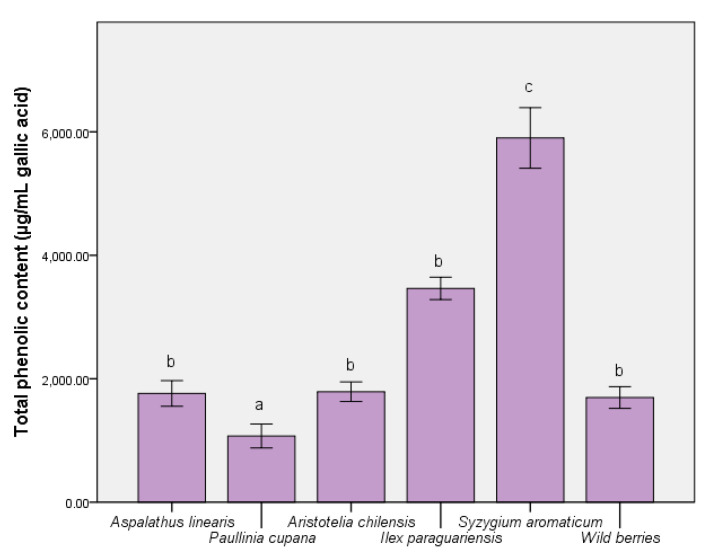
The total quantities of phenolic compounds in the extracts. Different letters in each column represent significant statistical differences (*p* ≤ 0.05) between extracts, n = 4.

**Figure 2:**
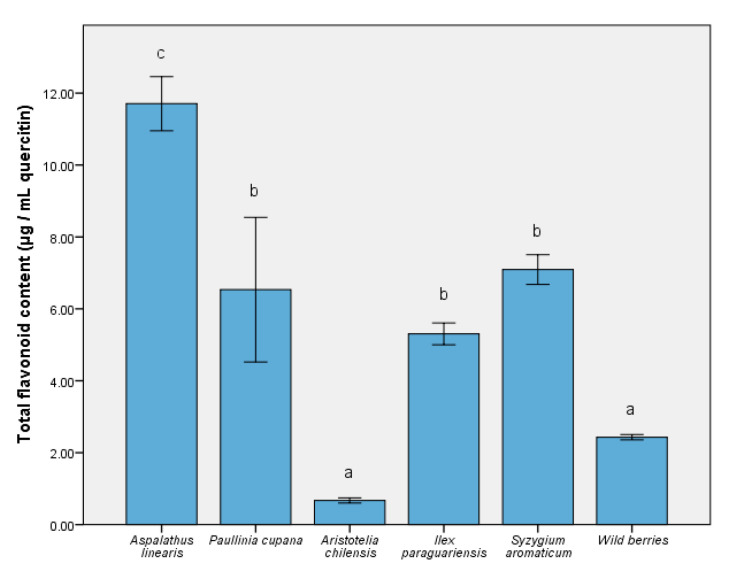
The total quantities of flavonoid compounds in the extracts. Different letters in each column represent significant statistical differences (*p* ≤ 0.05) between extracts, n = 4.

The ability of some nutraceuticals to reduce the physiological effects of oxidative stress was related to the distribution of bioactive compounds (like phenolic acids and flavonoids). Thus, Figure 1 depicts the level of major compounds responsible for expressing the biological effects. The complex distribution of nutraceutical effect molecules was evidenced in the *S. aromaticum* extract (5732.65 ± 87.59 µg/mL gallic acid equivalent), with significant differences from the remainder of the extracts (*p* ≤ 0.001). For *A. linearis* and maqui, the total phenolic quantity was similar to the wild berries sample. In contrast, the reduced quantity of phenolic compounds present when using *P. cupana* was inversely proportional to the expression of the antioxidant capacity in vitro [36]. In the case of the total flavonoid content measurement (Figure 2), the result was different; this result could be interpreted as an effect determined primarily by the tested samples and solvent. This was the case of *P. cupana* extract, which was similar to that of *S. aromaticum* (approximately 7.00 μg/mL, *p* ≤ 0.01) [37].

A similar pattern of major bioactive compounds was identified in the test samples, in which the main phenolic acids were chlorogenic, caffeic acids, and quercetin, as a flavonoidic compound. Table 1 reveals a high number of flavonoids and a varied distribution of kaempferol derivatives. The in vitro response was an expression of the distribution of these compounds [38], particularly for *S. aromaticum* and *I. paraguariensis*. In addition, the quercetin level for *S. aromaticum* was 4.67 ± 0.27 (*p* ≤ 0.001), greater than phenolic acids. Chlorogenic acid contained in the extract of *I. paraguariensis* showed a higher level compared to that in the remaining samples and control (*p* ≤ 0.001). The difference between the chemical quantification (Table 1) and assay results (Figure 1) was determined by the possible presence of other compounds in trace amounts that could not be determined. The solvent used (50% ethanol) determined a high solubility in water and the differences between these two assays were a result of this aspect. They were in quantities that did not directly affect the bioavailability results [2].

### 3.2. Determination of In Vitro Antioxidant Activities

**Figure 3:**
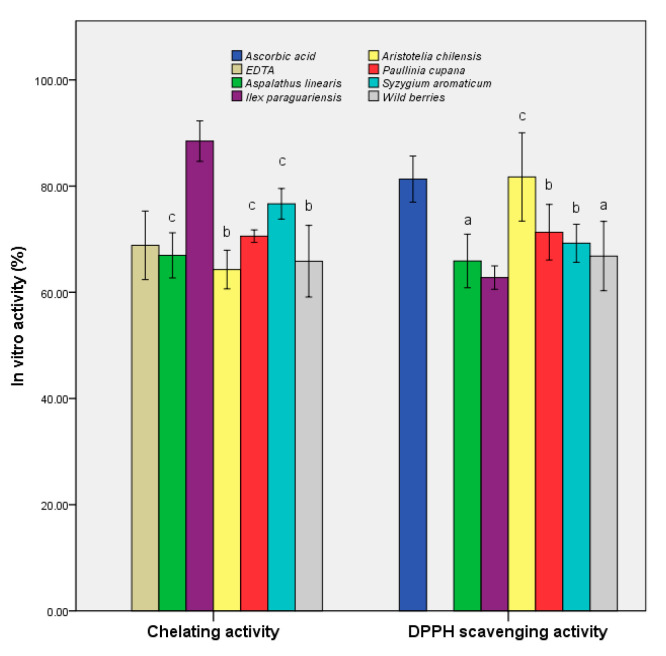
In vitro antioxidant activities of the samples. Different letters represent significant statistical differences (EDTA/ascorbic acid vs. samples; *p* ≤ 0.05), n = 3; EDTA/ascorbic acid was used as control.

We also sought to determine the antioxidant potential expressed as the DPPH-scavenging activity and chelation capacity (Figure 3). The maqui extract demonstrated the highest scavenging activity (81.71 ± 2.73%), which was similar to that of ascorbic acid (as a control), and 25% higher than that of the control sample (*p* ≤ 0.001). The remainder of the extracts showed values within ±10% of the second blank. The overall data suggested that maqui is a potential source of antioxidant compounds, the results being sustained by a previous study that demonstrated that the freeze-dried samples retained the highest quantity of bioactive compounds [39].

*I. paraguariensis* demonstrated a chelation capacity at least 25% higher than that of the control (88.47 ± 1.25%). The results demonstrated a prophylactic role in preventing the generation of free radicals in the case of administration of extracts as functional supplements. In addition, *A. linearis* exhibited a balance of the two properties (65.90 ± 1.65% for DPPH scavenging activity and 66.95 ± 1.39% for chelating activity), demonstrating pharmacological importance through multiple actions.

The exploitation of bioactive components in extracts also consists of their assimilation by biomass and the increase in the resistance to oxidative stress. This study aimed to use *S. boulardii* as a model for demonstrating the stability and resistance of eukaryotic yeast to the presence of free radicals.

### 3.3. Determination of Bioavailability Index

**Figure 4:**
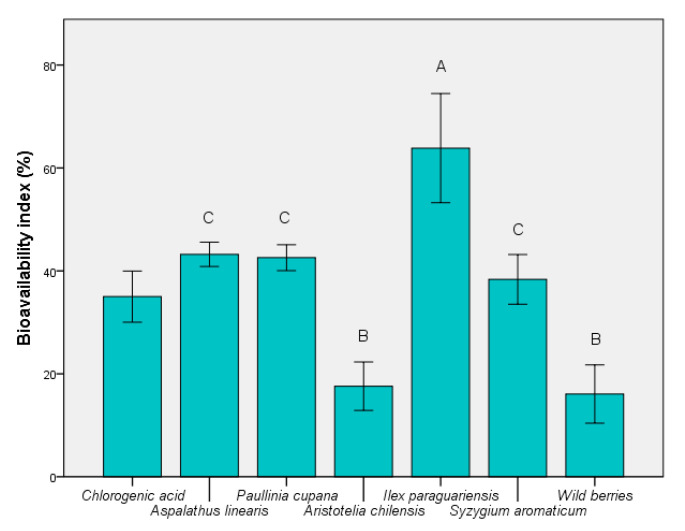
Bioavailability index of extracts based on *S. boulardii* in vivo model. Different letters represent significant statistical differences (chlorogenic acid vs. samples; *p* ≤ 0.05), n = 3; chlorogenic acid was used as control.

According to Figure 4, the highest BI was determined for *I. paraguariensis* extract, with an average value exceeding 60% (*p* ≤ 0.05). Wild berries showed the smallest value (*p* ≤ 0.01 vs. chlorogenic acid), although they are currently a widely-used product. The BI value was similar to the presence of maqui. It was possible to identify the correlation between the total presence of bioactive components, particularly chlorogenic acid. The stability of this compound in fermented medium is an advantage over the remainder of the unstable components (e.g., quercetin). This result showed that it cannot be used as a carbon source in the presence of probiotic strains, such as *S. boulardii*. These results support findings of recent studies that demonstrated the significant role of chlorogenic acid in the control of oxidative-stress-related causes [40].

This compound is important in its action in the colon because bioavailability depends on the metabolism exerted by the microbiota. It can be absorbed directly without affecting its chemical structure in case it directly reaches the colon. Otherwise, its derivatives (caffeic acid) are responsible for the antioxidant effects in vivo [41], and they can protect the eukaryotic cells in the presence of free radicals.

### 3.4. Determination of In Vivo Antioxidant Activities after the Assimilation Process

**Figure 5:**
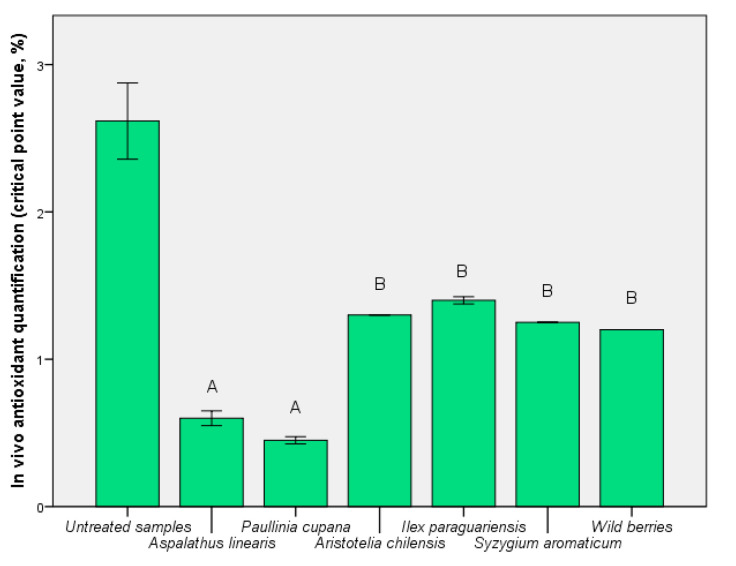
In vivo antioxidant activity before the bioassimilation process, but in the presence of extracts. Different letters represent significant statistical differences (untreated sample vs. samples; *p* ≤ 0.05), n = 3; untreated sample was used as control.

In Figure 5, the in vivo effect of the presence of the functional components is shown by the critical point value. The content of phenolic compounds showed a direct correlation, although for maqui, *S. aromaticum*, and *I. paraguariensis* the values were not significantly different (*p* < 0.01). A direct relationship between bioavailability index (Figure 4) and critical point value (Figure 5) was determined for *A. linearis* and *P. cupana*. For these tests, the results demonstrated a balance between exo- and endo-protection offered by these extracts. The exception was the use of *I. paraguariensis* extract which resulted in a highly-critical point, 1.40 ± 0.01% (*p* < 0.01 vs. untreated sample). This result demonstrated that a high quantity of phenolic compounds does not always cause in vivo protection against oxidative stress.

**Figure 6:**
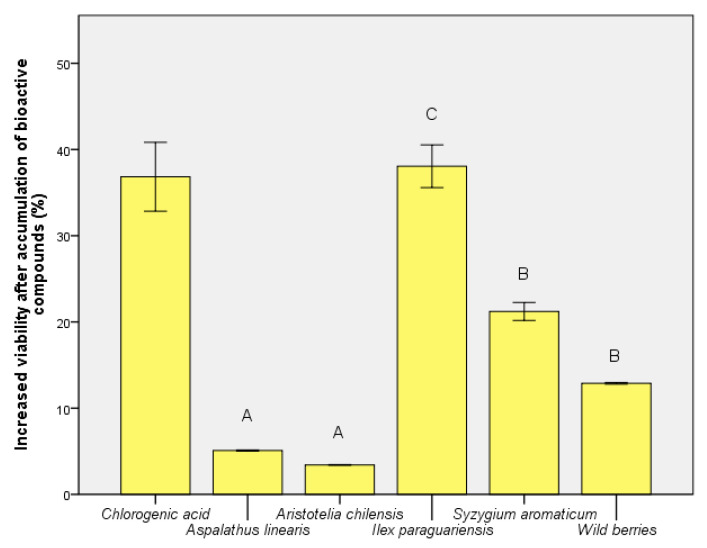
Increased viability of *S. boulardii* cells after bioactive compound assimilation. Different letters represent significant statistical differences (chlorogenic acid vs. samples; *p* ≤ 0.05), n = 3; chlorogenic acid was used as control.

After the bioactive compound assimilation, the improvement of viability is shown in Figure 6. The extract of *P. cupana* caused the loss of viability, and the result was considered to be determined by a pronounced antimicrobial effect or its use as a carbon source. Exo-protection calculated for *I. paraguariensis* could not be obtained even after fermentative assimilation. The result was similar to the control sample (chlorogenic acid) but ~60% higher compared to wild berries. The bioactive component was best assimilated for the extract of *S. aromaticum*, offering a viability increase of 21.21 ± 0.03% compared to the exo-protection phase. The result was interpreted as an effect of quercetin assimilation (Table 1).

### 3.5. Determination of Human Cell Viability

**Figure 7:**
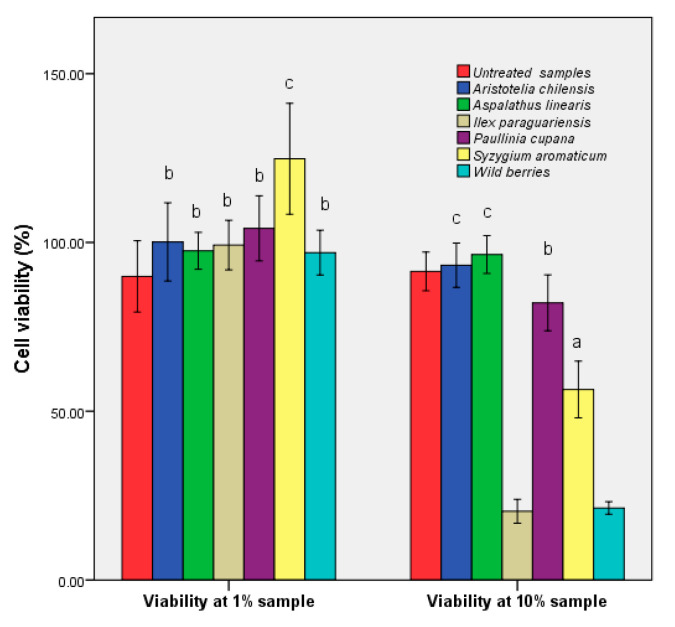
Evaluation of the HCT-8 cell viability via MTT assay in the presence of hydroalcoholic extracts. Different letters represent significant statistical differences (untreated sample vs. samples; *p* ≤ 0.05), n = 3; untreated sample was used as control.

The human epithelial cell viability in the presence of the plant extracts is shown in Figure 7. Exposure of the epithelial cells at low concentrations of hydroalcoolique plant extracts (1%) did not affect their viability; the exception was *S. aromaticum*, with an increase of cell viability of 10%. Instead, exposure of the cell line HCT-8 to higher concentrations of wild fruits and *I. paraguariensis* had a strong cytotoxic effect. The MTT assays measured the mitochondrial activity of cells. There was the possibility that *S. aromaticum* stimulated the cell enzyme activity. Other factors might have determined higher cell proliferation, such as slightly more cells were inoculated due to small pipetting errors, a favorable position in the plate, or just natural variations of cellular metabolism.

The cytotoxic activity was high in the extracts where the rutin was absent (Table 1), and the bioavailability showed average values. The minimum obtained was recorded in the samples that contained high amounts of phenolic acids, which could be understood as a form of exo-protection. Low flavonoid concentration determined specificity, which corresponded to previous studies performed on other tumor and nontumor cells [42]. For *A. linearis* the balanced distribution of the two major phenolic acids corresponded to a maximum cell number. The obtained results showed that the chemical composition was the main factor that influenced the cytotoxic activity. The HCT-8 cells morphology modifications after cultivation with 10% extracts are presented in Supplementary Figure S1.

**Figure 8:**
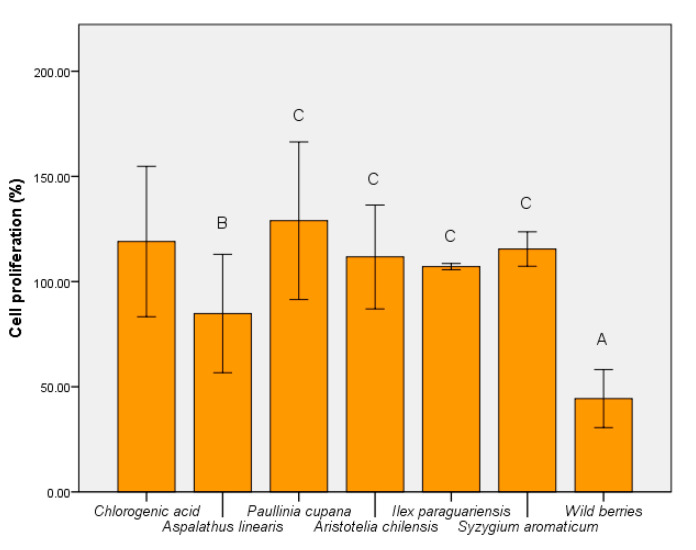
MTT assay of *S. boulardii* cells cultivated in the presence of hydroalcoholic extracts. Different letters represent significant statistical differences (chlorogenic acid vs. samples; *p* ≤ 0.05), n = 3; chlorogenic acid was used as control.

The metabolic activity of the strain *S. boulardii* was modulated uniquely by plant extracts. Wild berries, in addition to *A. linearis* had an inhibitory effect (Figure 8). In contrast, *P cupana* stimulated yeast cell metabolism (*p* ≤ 0.001). The effect of wild berries confirmed the cytotoxic effect shown at 10% concentration. This aspect demonstrated that treatments with wild berries and *I. paraguariensis* affect the metabolic activity of both yeast and human epithelial cell line HCT-8.

## 4. Discussion

The relationship between the degree of protection expressed in vitro and the possibility of any in vivo action was affected by the high bioassay variability as an expression of the biomass biological value. From the data obtained we can consider that such biomass (for example, for *I. paraguariensis*) provides protection from the formation of free radicals that cause oxidative stress. This study showed that bioaccessibility and bioavailability are indicators of the in vitro/in vivo relationship. The identification of the critical point of contact led to the definition of innovative mechanisms for the exploitation of bioactive components that can ultimately improve the bioaccessibility and bioavailability of the target compound. This aspect should be confirmed by further experiments. This in vivo indicator expressed the potential of the product (such as a functional extract, *I. paraguariensis*) to sustain the physiological mechanisms of protection against oxidative stress. This was an image of biological action after administration and could determine a real characterization of the bioactivity and bioavailability (Figure 4 and Figure 5).

The ability to assimilate yeast cells led to an understanding of the stability of the functional components and the importance that the pattern and the level of these compounds play in vivo. Thus, one of the perspectives that this study demonstrated was that increasing the concentration of a compound is not the essential factor; rather, it is the distribution of the whole spectrum of nutraceuticals in the tested product. This aspect explains the negative effects of the administration of green tea extracts [43] or the lack of a clinical result in many dietary supplements based on medicinally- or nutritionally-functional compounds [44]. The correlation between in vitro and in vivo data must be validated using laboratory models (yeasts) to demonstrate that a nutraceutical can also represent a pharmaceutical product. This link is one of the limiting points in the development of competitive products for personalized treatments (reducing inflammatory processes, for example) [2]. Such results would clinically validate the expansion of the nutraceutical industry to limit the action of the factors that determine degenerative pathologies.

The results shown in Figure 4 illustrate the high degree of absorption of the bioactive components of *I. paraguariensis* and *A. linearis*. The use of yeast was seen as an in vivo model to demonstrate the ability of the phenolic component for absorption after oral administration. This property was not correlated with cytotoxic capacity, demonstrating the biological versatility of chlorogenic acid as a bioactive compound. This study has contributed to the understanding of how the phytocomplex (particularly the active compounds) influences the eukaryotic cell response to oxidative stress. A chemical versatility that was controlled by the type of cells it interacted with was also demonstrated. The current study has shown that the phenolic pattern influences the same type of cells differently. The increase in the bioavailability index was correlated with poor cytotoxicity for *I. paraguariensis*.

Although the study did not take into account the influence of the physico-chemical factors during the gastrointestinal transit, it was considered that the remainder of the samples do not have a major influence as protectors in the action of oxidative stress. It can be assumed that the absorption in *S. boulardii* follows the same general principles as the absorption in the human intestinal lumen (e.g., molecular mass and degree of biotransformation of the phenolic component). Without eliminating comparative differences with multicellular organisms, strains belonging to the genus *Saccharomyces* are considered to be a model organism that can provide an overview of pharmacological applications that could be developed against oxidative stress [45].

Thus, the pattern of functional compounds and their molar ratio was considered the primary cause of bioavailability following prolonged exposure. The cytotoxic activity was high (i.e., over 90%) in extracts where rutin was absent (Table 1) because of possible bioconversion into rutin sulphate (metabolite; [46]). In addition, xenobiotic effects are possible as a secondary cause of blocking the cellular uptake process. Such effects were determined by secondary compounds, which act on the membrane transport mechanisms. In some studies, the effect of cell non-accumulation was considered as a positive effect (in the liver) because it did not involve increased cytotoxicity.

This study presented a series of in vitro and in vivo data that explain the exo- and endo-cellular mode of action of functional extracts. The biological effect showed specificity based on the profile of the bioactive compounds. It has been shown that different phenolic compounds have a characteristic ability to be assimilated, which explains the value of bioassimilation. The high capacity of incorporation of quercetin into eukaryotic cells increased the degree of protection against oxidative stress (Figure 5 and Figure 6). Phenolic acids (such as chlorogenic acid) did not significantly influence endo-oxidative protection. A positive correlation between phenolic compounds and in vitro activities was calculated for all extracts, but *I. paraguariensis* and *S. aromaticum* presented low values (*r* ≈ 0.3), similar to that of wild berries. A similar value was obtained in the case of cytotoxic activity of the same extracts and results observed for other berry samples, not only for representatives of the same berry. A high correlation value was calculated for *A. linearis* (*r* ≥ 0.5), and also for *P. cupana* and maqui (*r* ≥ 0.9) in the case of both in vitro activities. In contrast, in vivo antioxidant protection was directly correlated with the presence of the phenolic component (*r* ≥ 0.9) for all extracts.

This fact confirms that this acid is relevant as a non-pharmacological product because it has a non-invasive character in the prevention of degenerative pathologies [47]. For quercetin, the biological response was characteristic of a certain microbial species. Species of the same genus may have variability in the case of the bioavailability–biological effect. The results were confirmed by clinical studies that concluded that this compound showed differences at the individual level characterized by a pharmacologically-variable bioefficiency [48].

The bioavailability and expression of antioxidant action are dependent upon their primary action, and, by oral consumption, the function is profoundly affected in vivo [49]. Bioavailability via enriched biomass is a useful method for increasing the pharmacological value of *S. boulardii*, which enhances the assimilation of the active substance in the human colon. This is important because it increases the resistance of probiotic biomass to oxidative stress present in the case of dysbiosis. These products will maintain greater viability when interacting with the target microbiota and will be able to exert their effect for a longer time, an essential property in the microbial modulation process. In vivo delivery of compounds may be considered to improve blood concentration without considering biotransformations that may occur along the gastrointestinal tract. These aspects were supported by the present results, providing a lab mirror of the functional product that will have an in vivo effect.

## 5. Conclusions

In addition to the increase in the oxidative stress stability, bioassimilation and bioavailability of phenolic compounds define the in vivo complex action that phenolic compounds exert on interaction with eukaryotic cells. In conclusion, the use of *I. paraguariensis* extract increased the cellular capacity to protect against exogenous factors supporting oxidative stress compared with both the control sample and the assimilation of *I. paraguariensis*. The level and pattern of phenolic compounds expressed the antioxidant and cytotoxic activities. A correlation between bioactive compounds and different activities in vitro/in vivo was not obtained in the case of high levels because it was a species-specific relationship. An accurate understanding of bioassimilation and bioavailability processes will define the ability of probiotic biomasses to release functional components and will improve nutraceutical formulation.

## Figures and Tables

**Table 1:** Quantitative analysis of major bioactive compounds from the extracts.

Compounds (mg/100 mL Extract)	*Aspalathus linearis*	*Paullinia cupana*	*A. chilensis*	*Ilex paraguariensis*	*Syzygium aromaticum*	Wild Berries
Chlorogenic acid	0.21 ± 0.01 ^a^	0.60 ± 0.01 ^a^	0.04 ± 0.01 ^a^	14.38 ± 0.29 ^c^	0.06 ± 0.01 ^a^	0.37 ± 0.01 ^a^
Caffeic acid	0.11 ± 0.01 ^a^	0.01 ± 0.01 ^a^	0.005 ± 0.02 ^a^	0.19 ± 0.10 ^a^	0. 60 ± 0.08 ^b^	0.30 ± 0.01 ^b^
Rutin	nd	nd	nd	0.13 ± 0.01 ^a^	0.11 ± 0.01 ^b^	0.07 ± 0.08 ^b^
Quercetin	0.68 ± 0.04 ^c^	0.20 ± 0.01 ^a^	0.13 ± 0.01 ^a^	0.96 ± 0.04 ^c^	4.67 ± 0.27 ^c^	0.21 ± 0.02 ^b^
Kaempferol 7-glucoside	0.04 ± 0.01 ^c^	0.06 ± 0.05 ^b^	0.04 ± 0.01 ^b^	2.88 ± 0.30 ^c^	0.02 ± 0.01 ^a^	0.02 ± 0.01 ^a^
Kaempferol 3-galactoside	0.02 ± 0.03 ^b^	0.24 ± 0.01 ^a^	0.33 ± 0.05 ^c^	6.09 ± 0.29	1.07 ± 0.05 ^c^	0.01 ± 0.03 ^c^
Kaempferol 3-rutinoside	0.06 ± 0.02 ^a^	0.03 ± 0.05 ^a^	0.04 ± 0.02 ^a^	0.56 ± 0.41	0.10 ± 0.07 ^b^	0.03 ± 0.01 ^a^
Kaempferol 3-rhamnoside	0.01 ± 0.01 ^a^	0.02 ± 0.05 ^a^	nd	0.24 ± 0.03 ^b^	0.04 ± 0.08 ^c^	0.08 ± 0.05 ^b^
Myricetin 3-glucoside	0.95 ± 0.08 ^b^	0.11 ± 0.01 ^a^	0.09 ± 0.04 ^b^	0.03 ± 0.01 ^b^	0.40 ± 0.03 ^b^	0.002 ± 0.07 ^a^
Quercetin 3-rhamnoside	0.14 ± 0.01 ^a^	1.84 ± 0.04 ^c^	0.31 ± 0.09 ^b^	40.52 ± 0.93	0.37 ± 0.01 ^b^	0.24 ± 0.01 ^a^

Different letters represent significant statistical differences (*p* ≤ 0.05) between extracts, n = 3; nd—not detected.

## Supplementary Materials

The following are available online at https://www.mdpi.com/2304-8158/9/7/953/s1, Figure S1: HCT-8 cells’ morphology modifications after cultivation with 10% extracts, 200X.
