# Supplementary material for: The Bioactive Potential of Functional Products and Bioavailability of Phenolic Compounds
Source: Foods. 2020 Jul 18;9(7):953. doi: 10.3390/foods9070953 (PMC7404707; doi:10.3390/foods9070953)
Supplement: Supplementary file 1 [file foods-09-00953-s001.pdf]

Supplementary Material

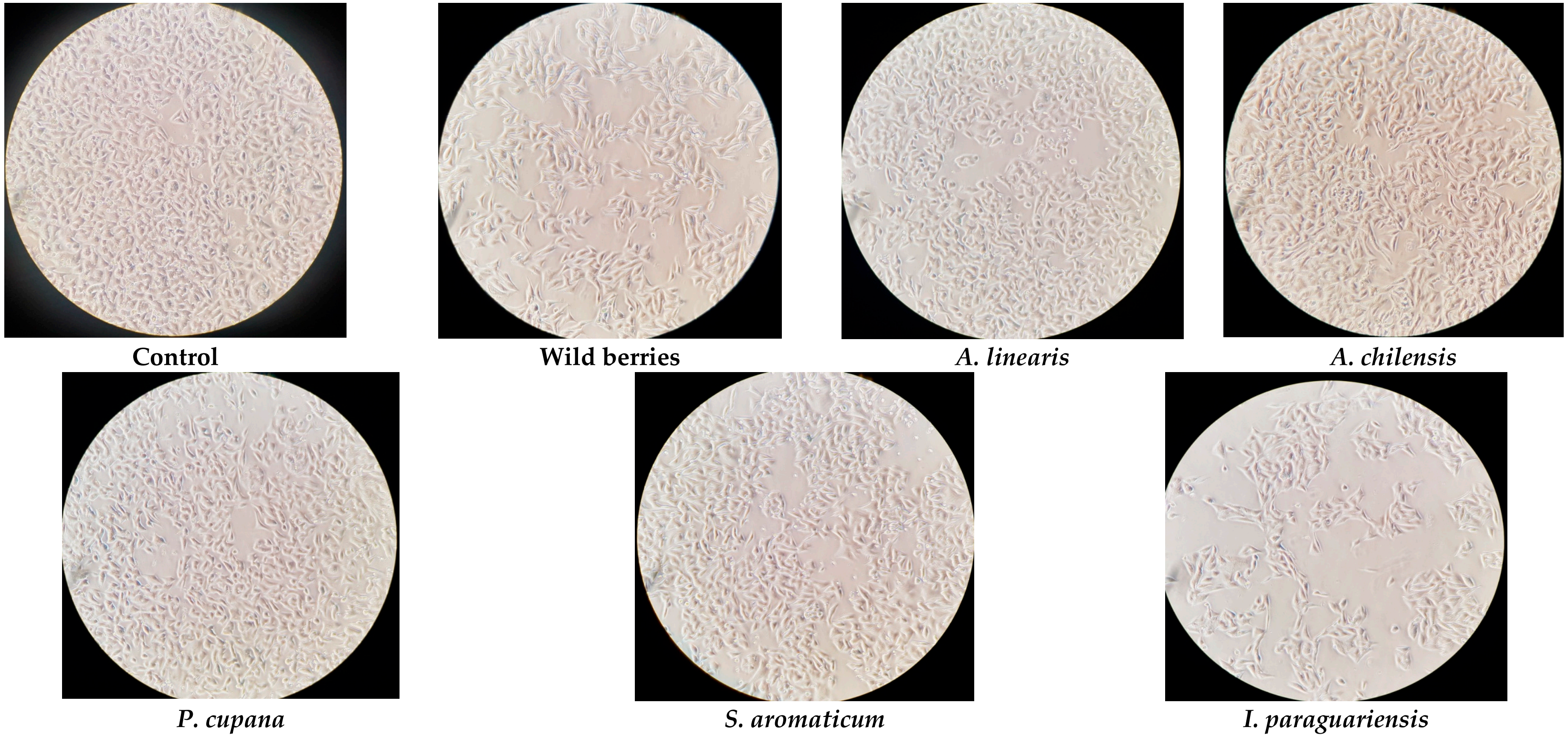

Supplementary Figure S1. HCT-8 cell morphology modifications after cultivation with 10% extracts, 200X.
